# Ancient horizontal transfer of transaldolase-like protein gene and its role in plant vascular development

**DOI:** 10.1111/nph.13183

**Published:** 2014-11-24

**Authors:** Zefeng Yang, Yong Zhou, Jinling Huang, Yunyun Hu, Enying Zhang, Zhengwen Xie, Sijia Ma, Yun Gao, Song Song, Chenwu Xu, Guohua Liang

**Affiliations:** 1 Jiangsu Key Laboratory of Crop Genetics and Physiology/Co-Innovation Center for Modern Production Technology of Grain Crops, Key Laboratory of Plant Functional Genomics of the Ministry of Education, Yangzhou UniversityYangzhou, 225009, China; 2 Department of Biology, East Carolina UniversityGreenville, NC, 27858, USA

**Keywords:** bacteria, horizontal gene transfer (HGT), land plants, rice (*Oryza sativa*), TAL-type transaldolase (*TAL*), vascular development

## Abstract

- A major event in land plant evolution is the origin of vascular tissues, which ensure the long-distance transport of water, nutrients and organic compounds. However, the molecular basis for the origin and evolution of plant vascular tissues remains largely unknown.
- Here, we investigate the evolution of the land plant TAL-type transaldolase (*TAL*) gene and its potential function in rice (*Oryza sativa*) based on phylogenetic analyses and transgenic experiments, respectively.
- *TAL* genes are only present in land plants and bacteria. Phylogenetic analyses suggest that land plant *TAL* genes are derived from Actinobacteria through an ancient horizontal gene transfer (HGT) event. Further evidence reveals that land plant *TAL* genes have undergone positive selection and gained several introns following its acquisition by the most recent common ancestor of land plants. Transgenic plant experiments show that rice *TAL* is specifically expressed in vascular tissues and that knockdown of *TAL* expression leads to changes in both the number and pattern of vascular bundles.
- Our findings show that the ancient HGT of *TAL* from bacteria probably plays an important role in plant vascular development and adaptation to land environments.

## Introduction

Horizontal gene transfer (HGT), also known as lateral gene transfer (LGT), refers to the transfer of genetic material between organisms with reproductive isolation. HGT plays an important role in the adaptive evolution of recipient lineages because the acquisition of novel genes may allow the recipient organism to explore new niches or to utilize new resources (Keeling & Palmer, 2008; Andersson, 2009). HGT is a major force driving the evolution of prokaryotes and leads to the spread of certain adaptive traits, such as antibiotic resistance and virulence (Nogueira *et al*., 2009; Palmer *et al*., 2010). Traditionally, HGT is thought to be frequent in prokaryotes and unicellular eukaryotes, but rare in multicellular eukaryotes, such as animals and plants (Huang & Gogarten, 2008; Keeling & Palmer, 2008; Keeling, 2009; Huang, 2013). However, recent investigations indicate that HGT also play important roles in the evolution of animals and plants (Richards *et al*., 2009; Dunning Hotopp, 2011; Yang *et al*., 2013). A genome-wide scan of the moss *Physcomitrella patens* revealed that 128 nuclear genes were acquired through HGT from prokaryotes, fungi, or viruses. These genes are involved in many plant-specific activities, including xylem formation, plant defense, nitrogen recycling, and the biosynthesis of starch, polyamines, hormones, and glutathione (Yue *et al*., 2012, 2013).

The pentose phosphate pathway (PPP), a key series of enzymatic reactions, is a process that generates NADPH and pentoses (five-carbon sugars) (Kruger & von Schaewen, 2003). There are two distinct branches in this pathway, one being the oxidative branch that leads to the production of NADPH, and the other the nonoxidative branch with roles in the synthesis of five-carbon sugars (Caillau & Paul Quick, 2005). In the nonoxidative branch of the PPP, transaldolase (TA) is a key enzyme that catalyzes the reversible transfer of a dihydroxyacetone group from fructose-6-phosphate to erythrose-4-phosphate, yielding sedoheptulose-7-phosphate and glyceraldehyde-3-phosphate. In addition, the roles of transaldolase, together with transketolase, also include a link between the glycolytic and PPP (Takayama *et al*., 1997; Caillau & Paul Quick, 2005).

Based on their conserved domains, the transaldolase family is divided into three subfamilies, including the transaldolase-like (TAL, cd00955), transaldolase_FSA (cd00956), and transaldolase_TalAB (cd00957) subfamilies. Although the TA subfamilies are well conserved in biochemical properties and the architecture of active sites, their overall sequence similarities are quite low (Caillau & Paul Quick, 2005; Samland *et al*., 2011). Land plants possess two types of TAs, including TAL and TalAB. The TalAB-type TA was proposed as the classical type, and members of this subfamily are ubiquitous to all three domains of life (i.e. archaea, bacteria, and eukaryotes), whereas the TAL-type TA is present only in land plants and bacteria. Two types of plant *TA* genes appear to be differentially expressed in response to environmental factors, suggesting that the TAL and TalAB isoforms have a nonoverlapping role in plant metabolism (Caillau & Paul Quick, 2005).

The ubiquity of the TAL-type TA in land plants suggests that its functions include a wide range of selectivity. In this study, the sequence similarity, phylogenetic relationships, and taxonomic distribution of the TAL-type *TA* genes were used to determine its origin in land plants. Our analyses revealed that the land plant *TAL* genes originated from an ancient HGT event from bacteria. Further studies with transgenic rice revealed that the *TAL* gene is required for rice vascular patterning.

## Materials and Methods

### Identification of *TAL* genes

To identify the genes encoding TAL-type TA in the land plants, the protein sequence of the *Arabidopsis* gene *At5g13420* was used as a query to search the Phytozome database (Goodstein *et al*., 2012). If a protein sequence satisfied *E* ≤ 10^−10^, it was selected as a candidate protein. Then, the CD-search tool in the Conserved Domain Database (CDD) of the National Center for Biotechnology Information (NCBI; Marchler-Bauer *et al*., 2013) was used to predict the transaldolase-like domain (cd00955). The newly identified TAL sequences detected in the land plants were used iteratively to search the respective sequence database. The deduced nucleotide and protein sequences of land plant *TAL* genes identified in this analysis were downloaded from the Phytozome database.

To identify the homologs of land plant *TAL* genes, BLAST searches against NCBI *nr*, dbEST, as well as JGI and other available eukaryotic genome databases (Supporting Information Table S1) were performed using land plant TAL proteins as queries. The sequences were further analyzed using an NCBI conserved domain search to confirm the presence of a transaldolase-like domain in the protein structure.

### Phylogenetic analyses

Protein sequence alignment was performed with Clustal X (Larkin *et al*., 2007) followed by visual inspection and manual refinement. The gaps and ambiguously aligned sites were removed manually. The program Modelgenerator (Keane *et al*., 2006) was used to identify the optimal model of protein substitution and rate heterogeneity. Phylogenetic analyses were performed with a maximum likelihood (ML) approach using PhyML version 3.0 (Guindon *et al*., 2010) and a neighbor-joining method using MEGA (Tamura *et al*., 2013). The ML phylogenetic analyses were conducted with the following parameters: JTT model, estimated proportion of invariable sites, four rate categories, estimated gamma distribution parameter, and optimized starting BIONJ tree. The JTT model was also used to construct the NJ trees. A total of 100 nonparametric bootstrap samplings were performed to estimate the support level for each internal branch for both the ML and NJ trees. The branch lengths and topologies of all phylogenies were calculated using PhyML. The phylogenetic trees were visualized using the *explorer* program in MEGA.

### Detection of positive selection

To test the selective pressure of the *TAL* genes during the long period of evolution in both land plants and Actinobacteria, the values of the *d*_N_ : *d*_S_ ratio (or *ω*) for two groups of *TAL* genes were calculated with the program *codeml* from PAML v4.4 (Yang, 2007). In this analysis, only Actinobacterial *TAL* genes located in the same branch as land plant homologs were used. Two phylogenetic trees for land plant *TAL* genes and their close homologs in Actinobacteria were reconstructed using PhyML (Guindon *et al*., 2010). The PAL2NAL program (Suyama *et al*., 2006) was used for the conversion of protein sequence alignment into the corresponding codon-based nucleotide alignment, which, in turn, was used for input into the *codeml* program in PAML. Here, we used three likelihood ratio tests (LRTs), M0 vs M3, M1a vs M2a, and M7 vs M8, to examine the selective pressure. The LRT for the comparison of M0 vs M3 was used to test the heterogeneity between the codon sites in *ω*, whereas the other two LRTs were used to detect the role of positive selection. For one LRT, twice the difference of the log likelihood of the two models was compared with the chi-squared (*χ*^2^) statistics, with the degrees of freedom equal to the difference in the number of parameters. In our analyses, the degrees of freedom are three for the M0/M3 test and two for the M1a/M2a and M7/M8 tests (Nielsen & Yang, 1998; Wong *et al*., 2004).

The improved branch-site model (Zhang *et al*., 2005) was also used to detect the role of positive selection that acted on the land plant *TAL* genes after HGT. In this model, the phylogeny is partitioned into foreground and background branches, with positive selection potentially occurring along the former. Here, the phylogenetic tree was generated using land plant and actinobacterial *TAL* genes, and the branch of land plants was used as the foreground. For this analysis, we compared the null hypothesis (*ω* fixed to 1) with the alternative hypothesis (free *ω*), to test whether positive selection acted on the evolution of land plant *TAL* genes. The Bayes empirical Bayes procedure in *codeml* (Yang *et al*., 2005) was used to calculate the posterior probability that each site was subject to positive selection in the foreground branch.

### RNA extraction and gene expression analysis

Total RNA was extracted from different tissues during the heading stage. RNA extraction was performed according the manufacturer's protocol for the RNA prep pure plant kit (Tiangen, Beijing, China). Approximately 1 μg total RNA from each sample was used for first-strand cDNA synthesis. Quantitative PCR was performed using the following gene-specific primer pairs: 5′-AGATACGAGGCTGTGATTGA-3′ and 5′-TCTTGGCACCTTTCTTGAC-3′ for the rice (*Oryza sativa* L.) *TAL* gene and 5′-GATGACCCAGATCATGTTTG-3′ and 5′-GGGCGATGTAGGAAAGC-3′ for *OsActin*, which was a control. Quantitative PCR was performed on a ViiA7 Real-Time System (Applied Biosystems, Foster City, CA, USA) with the SYBR Premix Ex Taq system (TaKaRa, Kusatsu City, Japan). Each set of experiments was repeated three times. The relative amount of the *TAL* transcript is presented as the 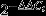 according to the Δ*C*_t_ method described in the real-time PCR Applications Guide.

### Transgenic analysis

To generate an RNAi construct, a 265 bp fragment of *TAL* was amplified from Nipponbare first-strand cDNA using the primers 5′-AAAGGATCCAGATACGAGGCTGTGATTGA-3′ containing a *Bam*HI recognition site and 5′-AAAACTAGTTCTTGGCACCTTTCTTGAC-3′ containing a *Spe*I recognition site. A hairpin structure with two inverted repeat fragments was then constructed and transferred into the plant binary vector p1301UbiNOS and expressed under the control of the maize ubiquitin promoter (Zhou *et al*., 2009).

For expression analysis, a 1138 bp genomic fragment upstream of the rice *TAL* gene translation start codon was PCR-amplified with the primers 5′-AAAGGATCCGGCAGATTTAGTAGAGCCATTTCTC-3′ and 5′-AAACCATGGTGACTCACATGATGGGGCTCCTG-3′. The DNA fragment was cloned into the *Bam*HI and *Nco*I sites of the vector pCAMBIA1301. The resulting plasmid was transformed into rice, and three independent *TAL* promoter-beta-glucuronidase (GUS) transgenic plants were analyzed using the GUS staining assay, as previously described (Zhou *et al*., 2009). All constructs were transformed by *Agrobacterium tumefaciens*-mediated transformation (Hiei *et al*., 1994).

### Morphological analysis

For histology, the fresh culm samples were removed from plants at the heading stage and then fixed in 2% glutaraldehyde, dehydrated in a graded ethanol series, and embedded in Spurr resin. Transverse sections were cut with an Ultracut EM UC7 (Leica, Solms, Germany), stained with 0.5% toluidine blue, and photographed using a DM1000 microscope system (Leica).

Leaf and stem morphometric analyses were performed at the heading stage. Leaf width and vascular pattern parameters were measured through the middle region of the flag leaf. The vascular pattern parameters of the leaves were measured in the dark-field microscopic digital images. The number of vascular bundles in the outer and inner rings of the stem was quantified in the digital microscopic images of the transverse sections through the middle region of the first and second internodes of the plants at the heading stage.

## Results

### *TAL* genes are widespread in land plants

**Fig 1:**
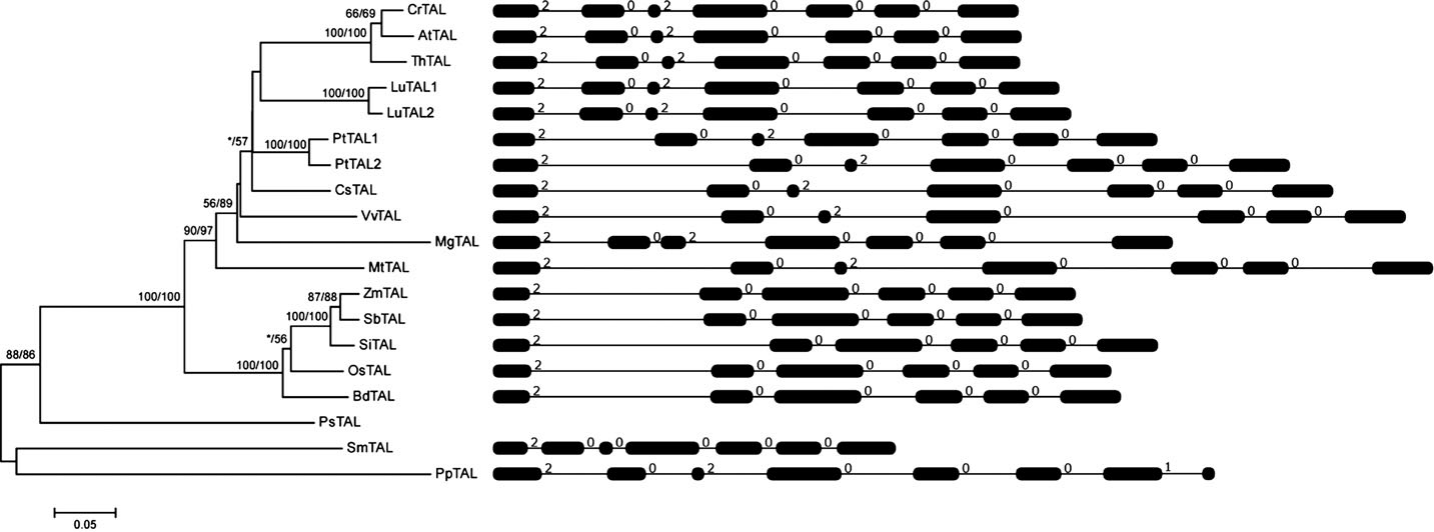
The phylogenetic tree of the land plant TAL-type transaldolase (*TAL*) genes and their exon/intron structures. The numbers above the branches represent the bootstrap values for the maximum likelihood and distance analyses, respectively. The asterisks indicate values < 50%. The exons are indicated by boxes, whereas introns are indicated by lines. The number above an intron indicates the phase.

Blast searches against the plant database revealed that each sequenced land plant genome contains at least one gene encoding TAL proteins. To explore the origin and evolutionary process of the land plant *TAL* genes, we characterized *TAL* genes from species representing the main lineages of land plants, including moss *P. patens*, lycophyte *Selaginella moellendorffii*, gymnosperm *Picea sitchensis*, five monocots and nine dicots (Table S2). We finally identified 19 *TAL* genes from the 17 genomes. Both the *Linum usitatissimum* and *Populus trichocarpa* genomes contained two *TAL* genes, whereas other sampled land plant genomes possessed only one *TAL* gene. Both the paralogous pairs were located on the terminal of the phylogenetic tree (Fig.1), indicating that they were formed through two distinct recent duplication events. We also found that both of these two paralogous pairs resulted from segmental duplication because there were highly conserved genes in the flanking regions for the two pairs of paralogous *TAL* genes in *L. usitatissimum* and *P. trichocarpa*.

Structural analysis of the *TAL* genes in land plants was performed by comparing the exon/intron organization. By comparing the CDS sequences with the corresponding genomic DNA sequences, we showed that the coding regions of all land plant *TAL* genes are interrupted by five to seven introns (Fig.1). The positions and phases of the majority of these introns in land plant *TAL* genes are conserved, demonstrating that the primary gene structure of this family was formed in the common ancestor of land plants. We also observed that the coding regions of *TAL* genes contained six introns in dicots, but only five introns in monocots. Through comparisons with homologs in *P. patens* and *S. moellendorffii*, we conclude that an intron in the middle of the monocot *TAL* genes was lost after the split between monocots and dicots.

### Land plant *TAL* gene was horizontally acquired from bacteria through an ancient transfer

The origin of land plants is a key event in the history of life and has led to dramatic changes in the environment and ecological systems of Earth. Evidence revealed that land plants evolved from charophycean green algae *c*. 480–490 million yr ago (Sanderson *et al*., 2004). Therefore, the majority of land plant genes must be vertically inherited. We searched the NCBI nr and JGI databases for the homologs of land plant TAL proteins. However, no homologs were found in any other eukaryote. The blast results also revealed that homologs of the land plant TAL proteins exist in bacteria only. In addition, the NCBI CDD also showed that genes encoding the TAL type TA proteins are found in land plants and bacteria only. The taxonomic distribution suggests that the evolution of the *TAL* gene is a result of an ancient HGT between bacteria and the ancestor of the land plants. Similarity searches and the presence of a conserved transaldolase-like domain (cd00955) indicate that the *TAL* genes are distributed widely in most bacterial lineages. The universality of the distribution in bacteria also suggests that this gene may have first emerged in bacteria.

**Fig 2:**
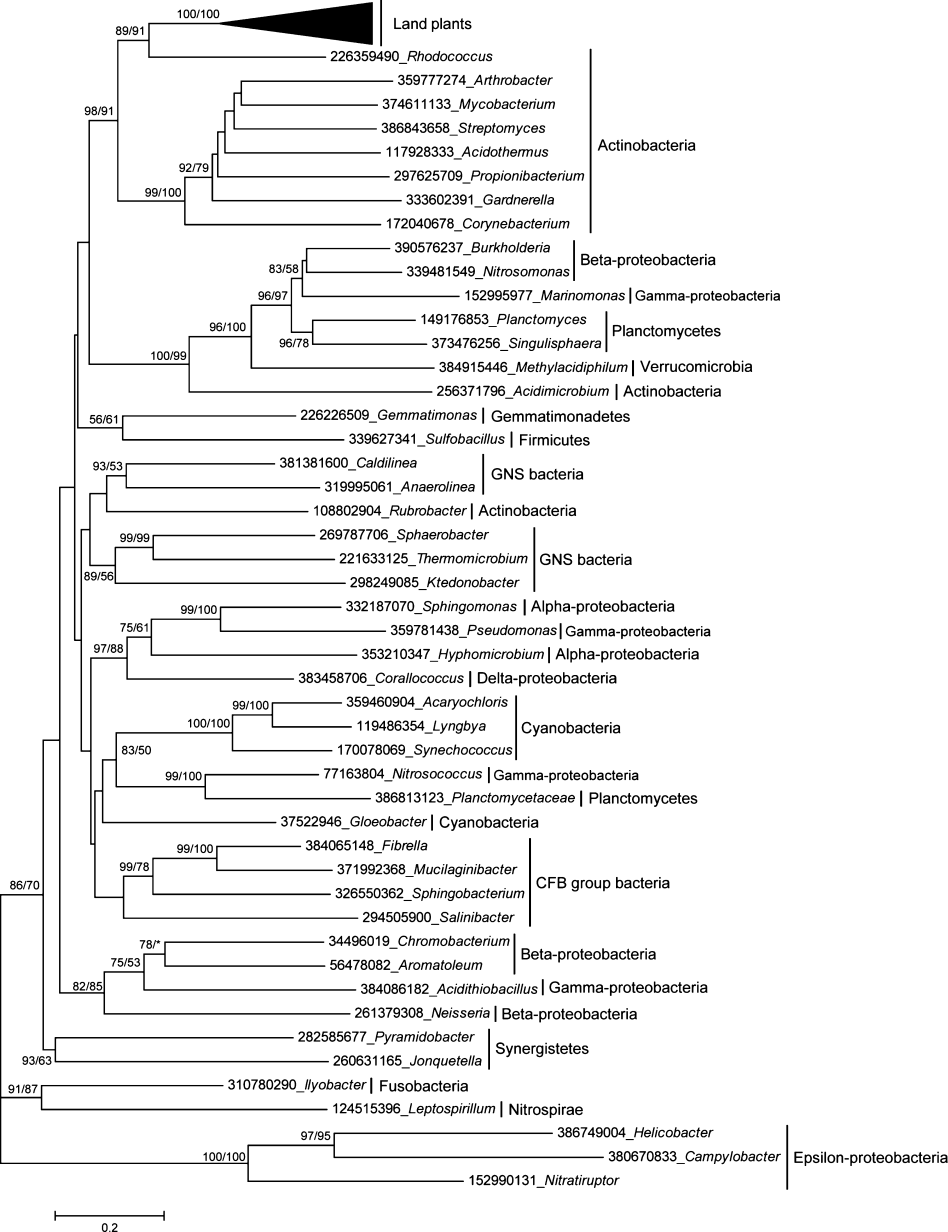
Phylogenetic analyses of TAL-type transaldolase (TAL) proteins. The numbers above the branches represent the bootstrap values for the maximum likelihood and distance analyses. All sequences were obtained from the National Center for Biotechnology Information (NCBI), except for those in the green plants, and each protein is indicated by the GI numbers in NCBI and its genus.

To determine the origin of the land plant *TAL* genes, we sampled representative taxonomical groups of cellular organisms in the nr database to build a phylogenetic tree (Fig.2). All selected proteins for the phylogenetic analyses possessed a transaldolase-like domain. Both the ML and distance phylogenetic trees showed a similar topology. Although the selected sequences cover the majority of primary bacterial lineages, in our analysis, the phylogenetic tree of the TAL proteins is not congruent with the species phylogeny, suggesting extensive gene losses, HGT, and selection within bacteria. In the phylogenetic tree, all the sampled land plant *TAL* genes form a single clade with high bootstrap support. The monophyly of land plant *TAL* orthologs strongly suggests that they have a single origin and are derived from a unique gene already present in the ancestor of land plants. In addition, we also observed that land plant *TAL* genes are in the clade of Actinobacteria genes, with high bootstrap support values for both methods. In addition, the close phylogenetic relationship of plant and Actinobacteria TALs is supported by the observation that land plant TALs have the highest similarity with their actinobacterial homologs. These findings demonstrate that land plant *TAL* genes originated from a single ancient HGT event from Actinobacteria before the separation of the land plant lineages.

### Positive selection facilitated the evolution of *TAL* genes in plants

The likelihood ratio tests of positive selection were applied using ML methods and the codon substitution models of Yang and colleagues (Nielsen & Yang, 1998; Yang *et al*., 2005; Yang, 2007). In this analysis, all the sampled land plant *TAL* genes and eight actinobacterial genes following to the same branch of plants were tested, respectively. First, we compared models M0 and M3 to determine whether there were *d*_N_ : *d*_S_ ratio variations for the codon positions for *TAL* genes both in land plants and Actinobacteria. Overall, both maximum likelihood estimates for the *d*_N_ : *d*_S_ values with model M0 were close to zero (Table S3), suggesting that purifying selection was the predominant force in the evolution of the *TAL* gene in both land plants and Actinobacteria. However, the log-likelihood differences between model M3 and M0 were statistically significant for both lineages, indicating that selective constraint level varied across amino acid positions. Secondly, the likelihood ratio tests to compare the data fit to models M2a vs M1a and M8 vs M7 were used to determine whether positive selection promoted the divergence of the *TAL* genes in both lineages. No amino acid site was found to be influenced by positive selection during the evolution of *TAL* genes either in land plants or in Actinobacteria. These results reveal that the primary constraints for the *TAL* gene in land plants after fixation from a horizontal gene transfer event was purifying selection.

To assess whether land plants are characterized by a different pattern of molecular evolution of the *TAL* genes compared with those in Actinobacteria, a new phylogeny was constructed using the TAL proteins in the branch of land plants and Actinobacteria. The branch of the new phylogeny leading to land plant *TAL* genes was classified as the foreground branch and those in Actinobacteria as the background branches. We found that the model that permitted a class of positively selected codons with *d*_N_ : *d*_S_ > 1 for the land plants branch had a significantly better fit to the data than the model in which this class of codon was restricted to *d*_N_ : *d*_S_ = 1 (Table 1). Because LRTs suggest that positive selection acted on the evolution of the *TAL* gene in land plants, the method of Bayes empirical Bayes (Yang *et al*., 2005) was used to evaluate the positively selected sites and their posterior probabilities. A total of 52 codons were identified with a > 50% posterior probability of *d*_N_ : *d*_S_ > 1 along the land plant branch. Of these, 11 amino acid sites have a 95% posterior probability of positive selection (Fig. S1). The most reasonable explanation for these results is that the *TAL* gene has undergone adaptive evolution in the ancestor of land plants during a short period of time after the HGT event, although the dominant force for the evolution in land plants of these amino acid sites is purifying selection.

**Table 1:** Parameters of the branch-site models used for the detection of positive selection

Model	Log_e_ *L*	Parameters
Null	−17 119.9710	*p*_0_ = 0.8158, *p*_1_ = 0.0466, *p*_2a_ = 0.1302, *p*_2b_ = 0.0074
		Background: *ω*_0_ = 0.0471, *ω*_1_ = 1.0000, *ω*_2a_ = 0.0471, *ω*_2b_ = 1.0000
		Foreground: *ω*_0_ = 0.0471, *ω*_1_ = 1.0000, *ω*_2a_ = 1.0000, *ω*_2b_ = 1.0000
Alternative	−17 112.9058**	*p*_0_ = 0.7947, *p*_1_ = 0.0455, *p*_2a_ = 0.1511, *p*_2b_ = 0.0087
		Background: *ω*_0_ = 0.0485, *ω*_1_ = 1.0000, *ω*_2a_ = 0.0485, *ω*_2b_ = 1.0000
		Foreground: *ω*_0_ = 0.0485, *ω*_1_ = 1.0000, *ω*_2a_ = 48.9623, *ω*_2b_ = 48.9623

**, *P* < 0.01.

### *TAL* gene is involved in rice vascular patterning

**Fig 3:**
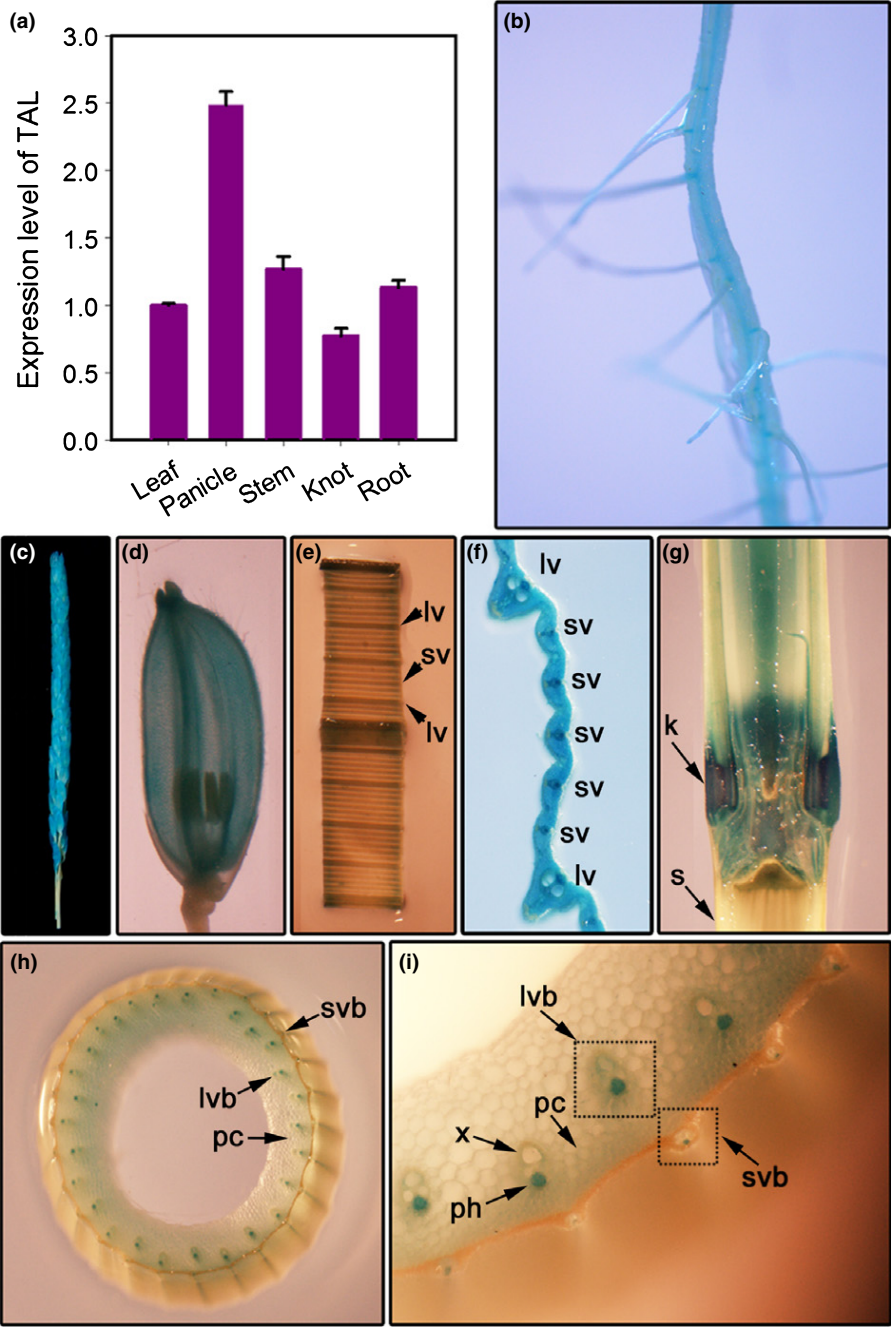
Expression analyses of the rice (*Oryza sativa*) TAL-type transaldolase (*TAL*) gene. (a) Quantitative reverse transcription polymerase chain reaction (qRT-PCR) analysis of rice *TAL* expression. Total RNA was isolated from the leaf, panicle, stem, knot, and root of wildtype plants at the heading stage. Amplification of the rice *Actin* gene was used as a control. Error bars represent + 1SE. (b) Beta-glucuronidase (GUS) activity in root. (c) GUS activity in panicle. (d) GUS activity in glume. (e) GUS activity in leaf at the heading stage. (f) Magnified image of the leaf in (e) showing the strong expression of *TAL* in developing vascular bundles of leaf. (g) GUS activity in knot and stem. (h) Transverse section of a third internode at the heading stage, showing the expression of *TAL* promoter-GUS in the vascular bundles of the stem. (i) Magnified image of the stem in (h) showing the strong expression of *TAL* in the phloem of vascular bundles. lv, large vein; sv, small vein; s, stem; k, knot; lvb, large vascular bundles; svb, small vascular bundles; pc, parenchyma cells; x, xylem; ph, phloem.

To ascertain the potential function of this horizontally acquired gene, the rice *TAL* gene was selected for functional detection. Quantitative PCR analysis suggested that rice *TAL* was expressed in organs such as the leaf, panicle, stem, knot, and root (Fig.3a). Our results also revealed that flowering panicles accumulated the *TAL* transcript at the highest level and also at the lowest part of the sheath at the heading stage. Furthermore, we generated transgenic rice plants expressing a fragment of the *TAL* promoter fused with the GUS reporter gene. Three independent TAL promoter-GUS transgenic plants were analyzed. All of them showed a similar expression pattern. *TAL*-GUS expression was observed with a pattern of strong tissue specificity (Fig.3b–i), and GUS activity was detected mainly in the vascular bundles of tissues, including the large and small veins of leaves (Fig.3e,f). In a cross-section of the stem at the heading stage, high GUS activities were detected in the phloem of vascular bundles (Fig.3h,i). The finding that *TAL* is preferentially expressed in vascular tissues indicated that this gene may be involved in rice vascular patterning.

**Fig 4:**
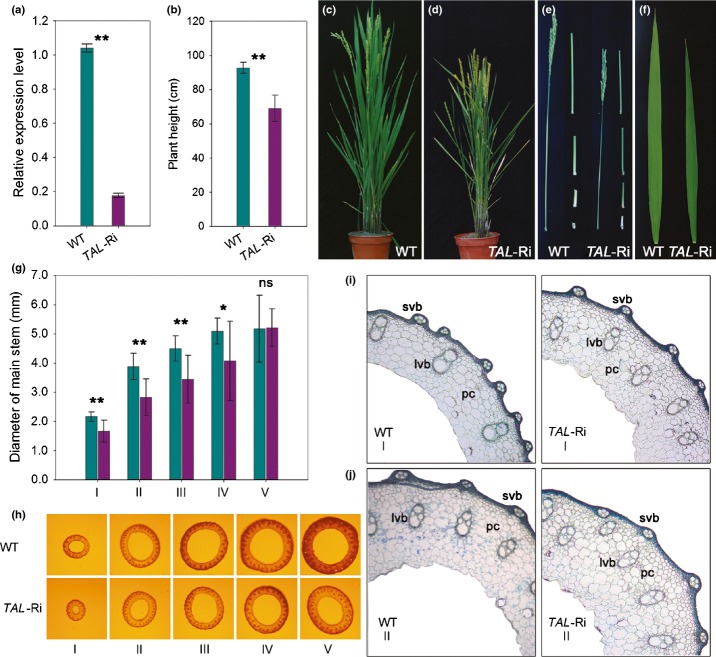
Phenotypic analysis of TAL-type transaldolase (*TAL*)-RNA interference (RNAi) in rice (*Oryza sativa*) plants. (a) Rice *TAL* gene expression levels in the leaves of wildtype (WT) and *TAL*-RNAi (*TAL*-Ri) plants during the heading stage. The *Actin* gene was amplified as the control. (b) Comparison of plant height between the WT and *TAL*-RNAi plants. (c, d) Plant phenotype of WT and *TAL*-RNAi plants. (e) Panicles and culms of WT and *TAL*-RNAi plants. (f) Flag leaves of WT and *TAL*-RNAi plants. (g) The diameter of axes from the first internode (I, panicle-neck internode) to the fifth internode (V, basal internode) of the main culms between the WT (green bars) and *TAL*-RNAi (purple bars) plants. (h) Cross-sections of the culm from the first internode to the fifth internode. (i) Transverse sections of the middle part of internode I of the WT and *TAL*-RNAi plants at the mature stage. (j) Transverse sections of the middle part of internode II of the WT and *TAL*-RNAi plants at the mature stage. lvb, large vascular bundles; pc, parenchyma cells; svb, small vascular bundles. The asterisks indicate the significance of differences as determined by Student's *t*-test as follows: **, *P* < 0.01; *, *P* < 0.05; ns, not significant. All data are means ± SE.

To further characterize the function of the rice *TAL* gene, we developed knockdown plants for the *TAL* gene in rice by introducing gene-specific RNA interference (RNAi) construct. We established 21 independent lines and grew them under normal conditions in the field. One line, *TAL*-RNAi, was used for further analysis. Quantitative PCR showed that the *TAL* transcript in leaves of the *TAL*-RNAi plant was significantly suppressed (Fig.4a). Leaves of the *TAL*-RNAi plants were shorter and narrower than those of the wildtype. The length and width of the *TAL*-RNAi flag leaves were *c*. 80.5 and 81.3% of the wildtype, respectively (Table S4, Fig.4f). In addition to the leaf shape, the *TAL*-RNAi plants also showed an altered culm elongation pattern. At maturity, the plant height of the *TAL*-RNAi was *c*. 71.0% of the wildtype (Fig.4b–e). We also compared the culm width between the *TAL*-RNAi and wildtype plants. As shown in Fig.4, the culm diameter of the uppermost four internodes (designated internodes I, II, III, and IV) of the *TAL*-RNAi was significantly smaller than the wildtype (Fig.4g,h). However, no significant difference in the culm width of the fifth internode was observed (Fig.4g,h).

A constant relationship between the leaf blade width and the longitudinal vein quantity in rice was observed in previous studies (Qi *et al*., 2008). A comparison of the flag leaves revealed that the total number of large veins and small veins was reduced in the *TAL*-RNAi plant (Table S4). This observation indicates that the rice *TAL* gene affects the formation of leaf veins, which may contribute to the narrow leaf morphology of the *TAL*-RNAi plant. The finding that *TAL* affects leaf venation prompted us to examine the vascular system in the culms of the *TAL*-RNAi plant. Although the vascular bundles were uniformly arranged in cross-sections of the *TAL*-RNAi internode I and II, the number of vascular bundles, particularly in the outer ring, significantly decreased (Table S4). In addition to the reduced number of vascular bundles, the *TAL*-RNAi plant also contained smaller and immature vascular bundles (Fig.4i,j). Furthermore, the average number of parenchyma cells in the region between the two adjacent large vascular bundles was reduced from approximately five in the wildtype to approximately three in the *TAL*-RNAi plant (Fig.4i,j). These findings reveal that *TAL* affects the vascular system in rice and that knockdown of *TAL* expression alters both the number and pattern of the vascular bundles.

## Discussion

Horizontal gene transfer is a major force driving the evolution of prokaryotes. Recent studies revealed that HGT also played a critical role in the transition of plants from aquatic to terrestrial environments (Yue *et al*., 2012, 2013). Plant genes with cyanobacterial and plastid-containing eukaryotic homologs as top hits are primarily derived from plastids, and many mitochondria-derived genes often have alpha-proteobacterial and other eukaryotic homologs as top hits (Huang & Gogarten, 2008). In this analysis, we found that no other eukaryotes contained genes encoding the TAL type TA and that land plant TAL showed the highest sequence percent identity with homologs from the Actinobacteria rather than alpha-proteobacteria and cyanobacteria. Therefore, under the assumption that the chance of the same gene being repeatedly transferred among different organismal groups is relatively low, the most parsimonious explanation is that the origin of the land plant *TAL* gene was the result of an ancient HGT event from Actinobacteria.

Generally, prokaryotic genes contain no introns. Although the *TAL* genes in bacteria have no introns, their homologs in land plants contain five to seven introns, and the majority of the intron/exon borders are highly conserved. It is postulated that the majority of these introns in land plant *TAL* genes arose through insertions shortly after the HGT event and before the divergence of the land plant lineages. Several other horizontally acquired eukaryotic genes were also found to have evolved introns through insertion events (Marcet-Houben & Gabaldon, 2010; Yang *et al*., 2013). In our analysis, we found that the dominant driving force for TAL gene evolution in both land plants and Actinobacteria was purifying selection, which contributes to functional stabilization. However, when we used the bacterial genes as a background, positive selection was found to significantly contribute to the evolution of the land plant *TAL* genes. Because positive selection is a major force underlying the adaptation of species to a new environment, the most reasonable explanation for these results is that the land plant *TAL* gene may have acquired some functional innovations through positive selection shortly after the HGT event and before the separation of major land plant lineages.

During their long period of evolution, land plants have adapted to terrestrial environments. To overcome the challenges of a dry environment and the lack of support for upright growth, plants evolved adaptive features, including cuticles, stomata, vascular tissue, gametangia, and seeds (Ligrone *et al*., 2012; Yue *et al*., 2012). The vascular system of plants consists of a network of continuous strands, the vascular bundles, which ensure the long-distance transport of water, nutrients, and organic compounds produced by photosynthesis in leaves (Ye, 2002). As a result of adaptive evolution, the origin of vascular tissues solved the problem of the long-distance transport of water and nutrients, thus enabling plants to gradually colonize the land. Studies revealed that HGT played important roles in the origin of genes associated with vascular development. It was proposed that the gene *VEP1* functions as a positive element required for vascular strand development in land plants (Jun *et al*., 2002). Systematic analysis found that the ancestor of land plants acquired the *VEP1* gene through an ancient HGT from bacteria (Tarrio *et al*., 2011). Lignin occupies the spaces in the cell wall between the cellulose, hemicellulose, and pectin components, particularly in the xylem tracheids, vessel elements, and sclereid cells. Phenylalanine ammonia-lyase (PAL), a critical enzyme of the general phenylpropanoid pathway that provides precursors for lignin monomer biosynthesis, was probably transferred from soil bacteria to fungi, and then secondarily routed to land plants (Emiliani *et al*., 2009). In this analysis, we not only demonstrated that the land plant *TAL* gene was acquired through an ancient HGT event, but also that this gene plays a significant role in the development of the plant vascular system using expression analysis and transgenic rice experiments.

Plant TA was first isolated from the potato, and further evidence shows that the plant TA is encoded by two distinct genes that evolved independently (Moehs *et al*., 1996; Caillau & Paul Quick, 2005). The detection of conserved domains revealed that one isoform is a TAL-type TA, such as PoTAL1 in tomato and At5g13420 in *Arabidopsis*, and the other is a TalAB-type, such as PoTAL2 and At1g12230. Only the TAL-type land plant TA is catalytically active when expressed as an *Escherichia coli* lysate (Caillau & Paul Quick, 2005). In addition, to cooperate with other OPPP enzymes to fine-tune the production of nonphotosynthetic redox power during the light phase, TA also has a specific role in plant defense mechanisms (Caillau & Paul Quick, 2005). However, green algae only possess TalAB-type TA, and no other eukaryotes contain genes encoding TAL-type TA, except land plants. In addition, different origination patterns were suggested for TAL and TalAB-type TAs because the low degree of sequence similarity between them. In this analysis, we found that the ancestor of land plants acquired the TAL-type TA from Actinobacteria through an ancient HGT event. This transfer probably confers an adaptive advantage for land plants because our transgenic experiments indicate that the rice *TAL* gene is involved in vascular patterning. Previous investigation of tomato TAL (ToTal1) abundance by western blot revealed that this enzyme localizes almost exclusively within vascular tissues (Caillau & Paul Quick, 2005), suggesting that the function of plant *TAL* gene in vascular patterning should be conserved in vascular plants, at least in angiosperm. However, we also found that the *TAL* gene is present in the genome *P. patens*, suggesting that the TAL-type TA may also have other functions in land plants in addition to its involvement in vascular patterning.

## Supporting Information

**Fig. S1** An alignment of the selected TAL protein sequences in land plants and Actinobacteria, showing the positively selected sites in land plants. **Table S1** List of the 50 eukaryotic complete genome sequences or ESTs used in this study, in addition to the NCBI nr database **Table S2** List of *TAL* genes in 17 representative land plant genomes **Table S3** The site-specific model parameters **Table S4** Morphometric analysis of wildtype and *TAL*-RNAi plants
