# Supplementary material for: Ancient horizontal transfer of transaldolase-like protein gene and its role in plant vascular development
Source: New Phytol. 2014 Nov 24;206(2):807–16. doi: 10.1111/nph.13183 (PMC4407918; doi:10.1111/nph.13183)
Supplement: Supplementary file 1 [file nph0206-0807-sd1.pdf]

Supporting Information Fig. S1 and Tables S1–S4

Table S1 List of the 50 eukaryotic complete genome sequences or ESTs used in this study, in addition to the NCBI *nr* database

Species	Lineage
Acanthamoeba castellanii	Amoebozoa
Antonospora locustae	Microsporidians
Aureococcus anophagefferens	Pelagophytes
Batrachochytrium dendrobatidis	Chytrids
Bigelowiella natans	Cercozoans
Blastocystis hominis	Stramenopiles
Brachionus plicatilis	Rotifers
Capitella sp.	Segmented worms
Capsaspora owczarzaki	Opisthokonta
Chlorella sp.	Green algae
Chlorella vulgaris	Green algae
Cyanidioschyzon merolae	Red algae
Cyanophora paradoxa	Glaucocystophyceae
Daphnia pulex	Crustaceans
Dictyostelium purpureum	Cellular slime molds
Emiliania huxleyi	Haptophytes
Euglena gracilis	Euglenoids
Glaucocystis nostochinearum	Glaucocystophyceae
Guillardia theta	Cryptomonads
Hartmannella vermiformis	Amoebozoa
Heterocapsa triquetra	Dinoflagellates
Hyperamoeba sp.	Amoebozoa
Isochrysis galbana	Haptophytes
Karenia brevis	Dinoflagellates
Karlodinium micrum	Dinoflagellates

Lottia gigantea	Gastropods
Malawimonas jakobiformis	Malawimonadidae
Mastigamoeba balamuthi	Amoebozoa
Monosiga ovata	Choanoflagellates
Naegleria gruberi	Heterolobosea
Paracercomonas marina	Cercozoans
Pavlova lutheri	Haptophytes
Physarum polycephalum	Plasmodial slime molds
Phytophthora ramorum	Oomycetes
Phytophthora sojae	Oomycetes
Polysphondylium pallidum	Cellular slime molds
Polytomella parva	Green algae
Porphyra yezoensis	Red algae
Prototheca wickerhamii	Green algae
Reclinomonas americana	Jakobida
Rhizopus oryzae	Fungi
Sawyeria marylandensis	Heterolobosea
Scenedesmus obliquus	Green algae
Seculamonas ecuadoriensis	Jakobida
Sphaeroforma arctica	Opisthokonta
Spironucleus vortens	Diplomonads
Streblomastix strix	Oxymonadida
Thalassiosira pseudonana	Diatoms
Trimastix pyriformis	Unclassified eukaryotes

Table S2 List of *TAL* genes in 17 representative land plant genomes

Lineage	Species	Name	Locus	Length	Intron	Chromosome	Location
Dicots	*Linum usitatissimum*	*LuTAL1*	Lus10043136	436	6	scaffold25	1384528–1386813
	Linum usitatissimum	*LuTAL2*	Lus10032613	436	6	scaffold140	1012925–1015257
	Populus trichocarpa	*PtTAL1*	POPTR_0003s16030	436	6	scaffold_3	15654057–15657021
	Populus trichocarpa	*PtTAL2*	POPTR_0001s12930	440	6	scaffold_1	10023678–10027179
	Medicago truncatula	*MtTAL*	Medtr7g006100	443	6	7	845072–850479
	Arabidopsis thaliana	*AtTAL*	AT5G13420	438	6	5	4301792–4304312
	Capsella rubella	*CrTAL*	Carubv10000972m	441	6	scaffold_6	4298572–4301148
	Thellungiella halophila	*ThTAL*	Thhalv10013572m	439	6	scaffold_2	4380727–4383435
	Citrus sinensis	*CsTAL*	orange1.1g036665m	440	6	scaffold00081	111594–114983
	Vitis vinifera	*VvTAL*	GSVIVG01001098001	441	6	1	22628856–22634048
	Mimulus guttatus	*MgTAL*	mgv1a006041m	460	6	scaffold_39	1460196–1462967
Monocots	*Sorghum bicolor*	*SbTAL*	Sb03g044500	429	5	chromosome_3	71853994–71856810
	Zea mays	*ZmTAL*	GRMZM2G134256	429	5	8	160390396–160393378
	Setaria italica	*SiTAL*	Si001538m	429	5	scaffold_5	45396389–45399497
	Oryza sativa	*OsTAL*	LOC_Os01g70170	432	5	1	40610513–40613463
	Brachypodium distachyon	*BdTAL*	Bradi2g59370	429	5	2	57090545–57093078
Gymnosperm	*Picea sitchensis*	*PsTAL*	ABR16241	444			
Fern	*Selaginella moellendorffii*	*SmTAL*	74909	423	5	scaffold_0	1351640–1353058
Moss	*Physcomitrella patens*	*PpTAL*	Pp1s159_68V6	438	7	scaffold_159	376836–382010

Table S3 The site-specific model parameters

Branch	Model	Log$_e$ L	Parameters	PSS under NEB[c]	PSS under BEB[d]
Land plants[a]	M0	-10086.8220	$\omega = 0.0608$	-	-
	M3	-9778.1731[**]	$p_0 = 0.5461, p_1 = 0.3549, p_2 = 0.0990$<br>$\omega_0 = 0.0025, \omega_1 = 0.0949, \omega_2 = 0.3612$	NAN	NAN
	M1a	-9973.4908	$p_0 = 0.9166, p_1 = 0.0834$<br>$\omega_0 = 0.0438, \omega_1 = 1.0000$	-	-
	M2a	-9973.4908	$p_0 = 0.9166, p_1 = 0.0834, p_2 = 0.0000$<br>$\omega_0 = 0.0438, \omega_1 = 1.0000, \omega_2 = 9.7249$	NAN	NAN
	M7	-9783.8626	$\beta(0.2728, 3.3243)$	-	-
	M8	-9596.8567	$\beta(0.2728, 3.3247)$<br>$p_1 = 0.9999, \omega = 1.0000$	NAN	NAN
Actinobacteria[b]	M0	-7869.1297	$\omega = 0.0022$	-	-

M3	-7576.9542 **	$p_0 = 0.2900, p_1 = 0.4343, p_2 = 0.2758$<br>$\omega_0 = 0.0001, \omega_1 = 0.0023, \omega_2 = 0.0087$	NAN	NAN
M1a	-7732.2492	$p_0 = 0.8041, p_1 = 0.1959$<br>$\omega_0 = 0.0343, \omega_1 = 1.0000$	-	-
M2a	-7732.2492	$p_0 = 0.8041, p_1 = 0.0086, p_2 = 0.1873$<br>$\omega_0 = 0.0343, \omega_1 = 1.0000, \omega_2 = 1.0000$	NAN	NAN
M7	-7581.9788	$\beta(0.5597, 99.0000)$	-	-
M8	-7581.9792	$\beta(0.5597, 99.0000)$<br>$p_1 = 0.0001, \omega = 3.2668$	NAN	NAN

[a]All the *TAL* genes list in Table S2 were used in this analysis. [b]Eight Actinobacteria *TAL* genes followed to the same branch of land plants on the phylogeny were used in this analysis. [c]Positively selected sites (PSS) under naive empirical Bayes (NEB) analysis; [d]positively selected sites (PSS) under Bayes empirical Bayes (BEB) analysis. NAN, not a number.

Table S4 Morphometric analysis of wild-type and *TAL*-RNAi plants

Organ	Wild-type	TAL-Ri
Leaf		
Flag leaf length (cm)	26.1 ± 4.9 (7)	21.0 ± 3.4 (9)*
Flag leaf width (cm)	1.6 ± 0.1 (7)	1.3 ± 0.3 (9)**
Number of large veins of flag leaf	13.6 ± 1.5 (7)	11.9 ± 3.6 (9)*
Number of small veins of flag leaf	49.4 ± 3.6 (7)	34.9 ± 5.9 (9)**
Stem		
Number of large vascular bundles of internode I	14.0 ± 0.8 (7)	10.0 ± 3.8 (9)**
Number of small vascular bundles of internode I	30.0 ± 8.6 (7)	22.0 ± 4.3 (9)*
Number of large vascular bundles of internode II	30.7 ± 1.4 (7)	26.0 ± 4.2 (9)**
Number of small vascular bundles of internode II	30.9 ± 0.9 (7)	25.8 ± 4.3 (9)**

The flag leaf, internode I and internode II morphometric analyses were carried out at heading stage. Results represent means ± SD of populations of the size indicated in parentheses. Asterisks indicate the significance of differences between wild-type and *TAL*-RNAi as determined by Student's *t* test: **, P = 0.01 level; *, P = 0.05 level.

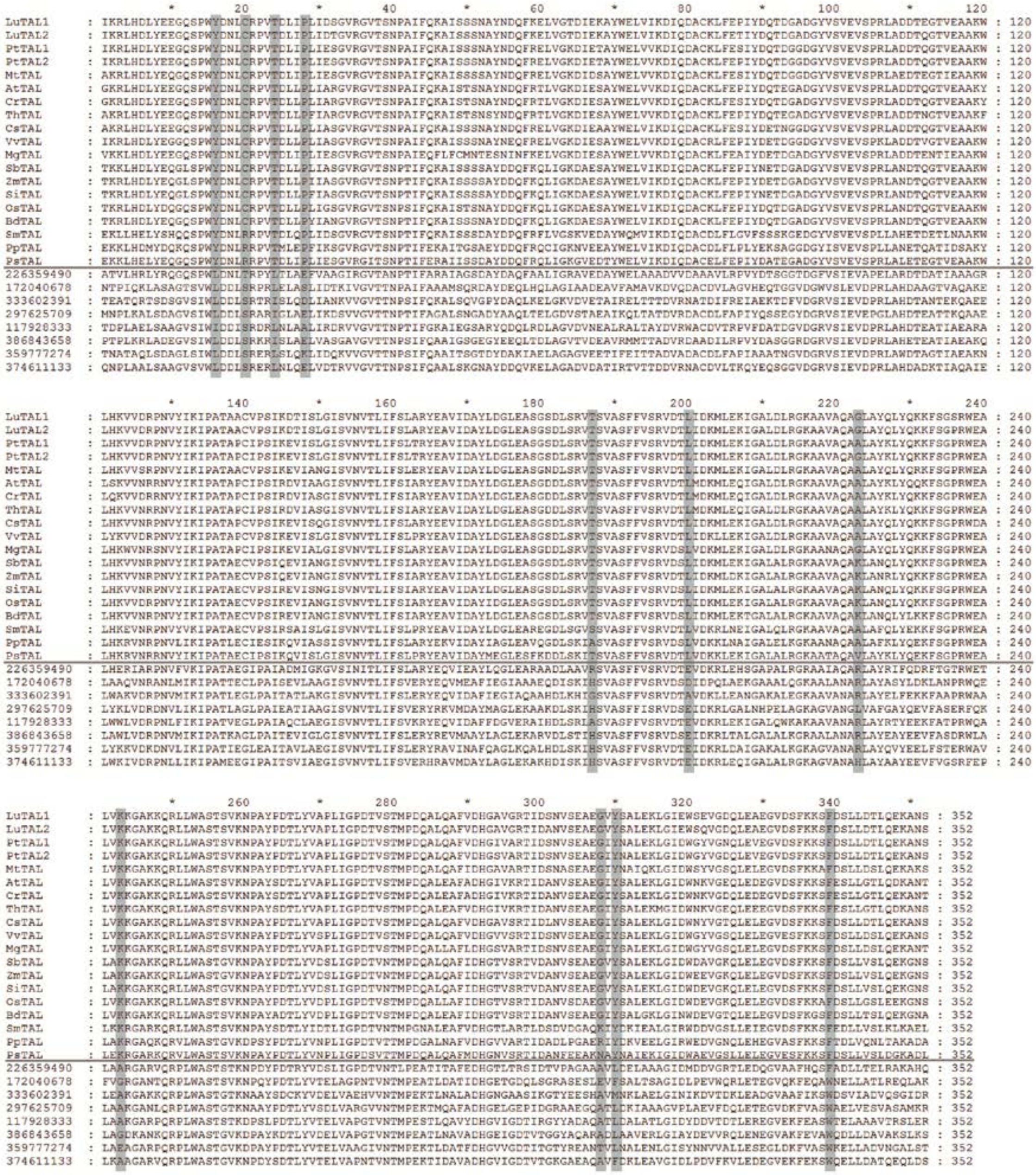

Fig. S1 An alignment of the selected TAL protein sequences in land plants and Actinobacteria, showing the positively selected sites in land plants. The positively selected sites are indicated by deckling.
